# The Prognostic Value of Anticholinergic Burden Measures in Relation to Mortality in Older Individuals: A Systematic Review and Meta-Analysis

**DOI:** 10.3389/fphar.2020.00570

**Published:** 2020-04-29

**Authors:** Katherine Graves-Morris, Carrie Stewart, Roy L. Soiza, Martin Taylor-Rowan, Terence J. Quinn, Yoon K. Loke, Phyo Kyaw Myint

**Affiliations:** ^1^Ageing Clinical and Experimental Research (ACER) Group, Institute of Applied Health Sciences, University of Aberdeen, Aberdeen, United Kingdom; ^2^Aberdeen Royal Infirmary, NHS Grampian, Aberdeen, United Kingdom; ^3^Institute of Cardiovascular and Medical Sciences, University of Glasgow, Glasgow, United Kingdom; ^4^Norwich Medical School, University of East Anglia, Norwich, United Kingdom

**Keywords:** anticholinergics, adverse outcomes, prognostic study, older adults, measurement scales

## Abstract

**Background:**

Greater anticholinergic burden (ACB) increases the risk of mortality in older individuals, yet the strength of this association varies between studies. One possible explanation for this variance is the use of different approaches to quantify ACB. This systematic review (PROSPERO number CRD42019115918) assessed the prognostic utility of ACB-specific measures on mortality in older individuals.

**Methods:**

Multiple cross-disciplinary databases were searched from 2006–2018. Observational studies assessing the association between ACB and mortality utilizing ≥1 ACB measure, involving persons aged ≥65 years were included. Screening and data extraction were performed by two independent reviewers, with disagreements resolved by a third independent reviewer. Risk of bias and quality of evidence were assessed using Quality in Prognosis Studies (QUIPS) and Grading of Recommendations, Assessment, Development, and Evaluations (GRADE) criteria. Meta-analysis was conducted where appropriate.

**Results:**

Of 19,224 titles, 20 articles describing 18 cohort studies involving 498,056 older individuals were eligible. Eight anticholinergic-specific measures were identified; the Anticholinergic Cognitive Burden Scale (ACBS, n=9) and Anticholinergic Risk scale (ARS, n=8) were most frequently reported. The evidence base was of poor quality, with moderate to high risk of bias. Meta-analysis showed increased mortality risk.

**Conclusions:**

There was a modest association between some ACB measures and mortality, with most evidence derived from the ACBS. Studies comparing different measures within the same population were lacking. Analysis was limited by poor generalizability between studies, specifically regarding heterogeneity in methodology and reporting, as well as high risk of bias for most studies in the evidence base.

## Introduction

The cumulative use of anticholinergics, termed anticholinergic burden (ACB), has been highlighted as one problematic area of polypharmacy in older populations (Hilmer et al., 2007; Salahudeen et al., 2015). It is estimated that 20–50% of older persons have been prescribed at least one medication with anticholinergic activity (Campbell et al., 2009), with increases in prescribing rates and ACB as individuals age (Lu et al., 2015). While anticholinergics are useful in conditions such as depression, incontinence, Parkinson’s disease, and gastrointestinal disorders, their broad range of action on the central and peripheral nervous systems can result in significant side-effects. Central side-effects include sedation, confusion and delirium (Rudolph et al., 2008; Fox et al., 2014). Peripheral side-effects include dry mouth and eyes, tachycardia, and constipation (Rudolph et al., 2008; Fox et al., 2014). Additionally, concerns have been raised as to the potential of anticholinergics to exacerbate physical and mental decline, especially regarding dementia development (Fox et al., 2014; Myint et al., 2014; Salahudeen et al., 2015). Consequently, various anticholinergics have been listed as potentially inappropriate medications for use in older individuals, as indicated by the Beers Criteria^®^ (The American Geriatrics Society Beers Criteria® Update Expert Panel et al., 2019) and STOPP criteria (O’Mahony et al., 2010).

One risk suggested to be associated with greater ACB amongst older individuals is mortality, although the significance and strength of this association varies between studies (Fox et al., 2014). One explanation may lie in how ACB was quantified. To date, multiple measures exist to assess ACB but differences are apparent between measures in terms of which medications are included and the level of anticholinergic potency attributed to individual medications (Bostock et al., 2013; Salahudeen et al., 2015). For example, clomipramine is assessed as having “high” potency according to Boustani et al. (2008) and Carnahan et al. (2006), yet this drug is not included in the analysis by Rudolph et al. (2008). Drug availability and formularies between countries can also impact the ability of measures, typically developed with the drug formularies of one country, to be applied internationally (Salahudeen et al., 2015). Currently, there is no evidence to support the decision to use or not use one ACB measure above another. Improving this understanding will allow informed ACB measure choices to be made and enhance the quality of future research. Therefore, this systematic review aims to describe the association of individual ACB measures with mortality in older adults.

## Methods

This systematic review, followed Preferred Reporting Items for Systematic Reviews and Meta-Analyses (PRISMA) guidance (www.prisma-statement.org; see **Supplementary Table 2** for PRISMA checklist) and was registered with PROSPERO (www.crd.york.ac.uk/prospero), registration number CRD42019115918).

### Literature Search Strategy

MEDLINE (Ovid), EMBASE (Ovid), PsycINFO (Ovid), and CINAHL (EBSCO) were searched using a comprehensive search strategy, adapted to suit each specific database. Study searches were undertaken on 16th November 2018. The validated search filter utilized to identify prognostic studies was in line with the recommendations of Geersing et al. (2012). Both MeSH and text/key words were included. Studies published between 1st January 2006 and 16th November 2018 were included. The initial timepoint of 2006 was used as cumulative ACB was first described during this period. Reference lists and citation checks of identified eligible studies were reviewed for further eligible studies. The search strategy is reported in **Supplementary Table 1**.

### Study Inclusion Criteria

Studies were deemed eligible if they were in accordance with the following criteria:

- Prospective and retrospective observational studies, for example longitudinal cohorts and case-control studies from any setting (e.g., community/primary care/general practice, secondary care/hospital/acute care, nursing homes/homes).
- Mean study participant age was ≥65 years.
- Human studies investigating the total exposure of anticholinergic burden by use of ACB-specific measure. The comparator required was non-users of anticholinergic medications and outcome of interest was mortality.

### Study Exclusion Criteria

Studies were excluded if:

- They were systematic reviews, randomized control trials, cross-sectional studies, opinion/editorial articles, qualitative studies, or animal studies.
- Study mean age was <65 years.
- Non-anticholinergic specific measures (e.g., BEERS criteria, Drug Burden Index_(total)_) were used
- Specific anticholinergic medications or groups of medications (e.g., bladder antispasmodics) were assessed and not total anticholinergic exposure.

### Study Selection Process

Studies identified through database searches were entered RefWorks (ProQuest LLC, Ann Arbor, MI, USA) for bibliographic management to remove duplicates. Studies were transferred to Covidence systematic review software ^©^2019 (Veritas Health Innovation Ltd., www.covidence.org) for screening, whereafter 13,197 studies remained after duplicate removal. Titles and abstracts of records were screened by two of three independent reviewers (shared between CS, KY, MK) to determine whether inclusion criteria were met. For a title to be excluded, two independent reviewers had to agree on exclusion; where disagreement occurred, a third independent reviewer (TQ) would aid resolution. Thereafter, full texts of potentially eligible studies (n=119) were obtained and further independently examined by two of three reviewers (CS, KY, MK) against the inclusion criteria to determine eligibility. Study authors were contacted where full texts could not be sourced. Again, a third independent reviewer (TQ) resolved any disagreements regarding exclusion. Included studies had their citations reviewed through PubMed and reference lists hand searched to check for further eligible studies. Reference lists and citations of recent seminal articles (Salahudeen et al., 2015) were also searched.

### Data Collection, Extraction, and Analysis

A data extraction template was developed in accordance with guidance by the Cochrane Prognostic Review Group framework (https://methods.cochrane.org/prognosis/our-publications) requirements for data. Key data extracted included study characteristics (e.g., publication date, country, study setting), population descriptives, ACB measure(s) used, types of data (e.g., continuous, ordinal), the timing of predictor, and outcome variables, covariates adjusted for, statistical plan and results. Data was extracted by two of three independent reviewers (KG, CS, MT); any disagreements were resolved by a third independent reviewer (RS). Thereafter, data were transferred to a Microsoft Excel 2016 (https://products.office.com/en-gb/excel) sheet and imported to Comprehensive Meta-Analysis (CMA) (v3.3.070 Biostat, Englewood, USA; https://www.meta-analysis.com/) for analysis, with forest plots produced where appropriate. All meta-analysis data presented compared ACB = 0 with ACB >0.

### Risk of Bias Assessment—QUIPS

The quality and risk of bias of included studies was assessed by the Quality in Prognosis Studies tool, developed by the Cochrane Prognosis Methods Group (QUIPS, available: https://methods.cochrane.org/sites/methods.cochrane.org.prognosis/files/public/uploads/QUIPS%20tool.pdf). The QUIPS tool comprises of six areas: study participation, attrition, prognostic measurement, outcome measurement, study confounding, and statistical analysis. The wording of the QUIPS anchoring statement was modified to be suitable for the current review question as recommended. Any published baseline measure of ACB was acceptable and adjusting for a minimum set of confounders (age, sex, and any measure of comorbidity burden) was considered adequate for assessing quality of statistical analysis. Risk of bias assessments were carried out by two of three reviewers (KG, CS, MT). Any discrepancies were resolved by a third independent reviewer (RS). The summary of the QUIPS risk of bias assessment can be found in **Figure 4**.

### Strategy for Data Synthesis

Data were analyzed and split into two primary groups: hazard ratio and odds ratio data. Data were further stratified according to the ACB measurement tool utilized and whether these data were continuous or ordinal. Where possible, analyses were further stratified by strength of ACB exposure into “high” or “low,” though precise definitions varied by study and were reported individually for each meta-analysis (see **Table 3** for definitions of “high” and “low” categories within each study). Meta-analyses were undertaken on comparable datasets. 95% confidence intervals (CIs) were utilized. CMA was used to facilitate the production of meta-analyses and forest plots. Random effects models were used.

If multiple results were provided in the same study, the most appropriate result was chosen to be included in the meta-analyses by two researchers (CS and KG) so as not to duplicate the same study population within one meta-analysis (see results). For Lattanzio 2018 A (Lattanzio et al., 2018a) and Lattanzio 2018 B (Lattanzio et al., 2018b), one cohort was used for both studies. Again, to avoid cohort duplication, data assessing ACB on the cohort as a whole (Lattanzio 2018 A (Lattanzio et al., 2018a)) were chosen as more generalizable than data produced after stratification of individuals by Basic Activities of Daily Living (BADL) scores.

Where available, data adjusted for covariates (e.g., age, sex, comorbidities) and/or temporal changes in ACB were given priority over unadjusted, baseline ACB-only data. For studies which had multiple follow-up periods, the follow up period most comparable to the other studies within the review was chosen. Where data were considered too heterogenous to be included in any meta-analysis, they were analyzed narratively.

### Quality Assessment—GRADE

The quality of the body of evidence collected throughout this systematic review was assessed with the GRADE tool. The GRADE tool assesses the quality of evidence for given outcomes of studies, as opposed to assessing the evidence from individual studies (Guyatt et al., 2008). Evidence quality was assessed by two researchers (CS, KG) across seven criteria; study limitations, inconsistency, indirectness, imprecision, publication bias, effect size and dose-effect. Subsequently, studies were upgraded or downgraded in relation to the seven criteria and a final decision was made as to the quality of evidence (ranging from high quality to very low quality). This process was adapted for prognostic review studies in accordance with the recommendations laid out by Huguet et al. (2013).

## Results

**Figure 1:**
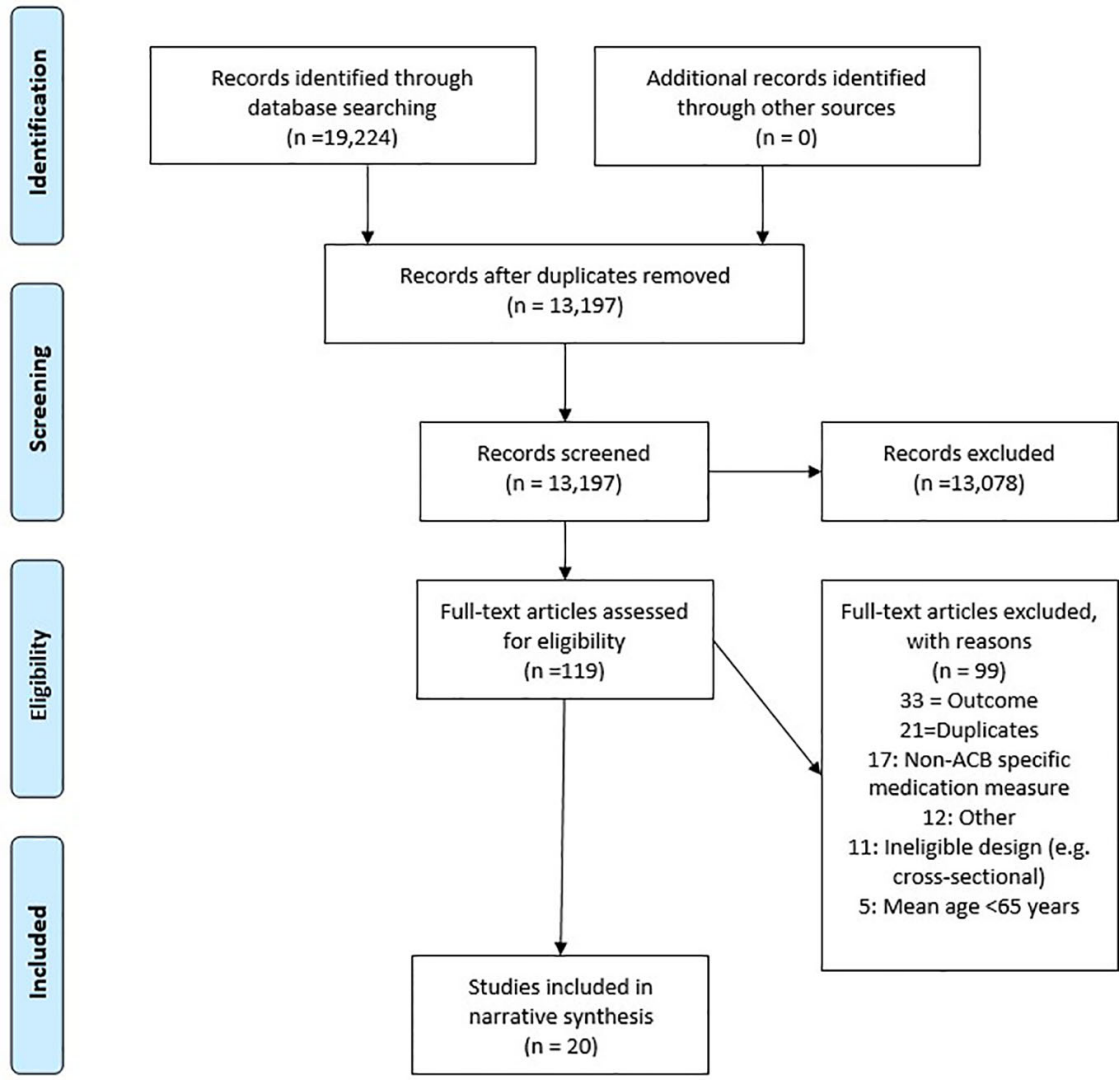
Preferred Reporting Items for Systematic Reviews and Meta-Analyses (PRISMA) flowchart depicting the study screening and selection process.

Twenty papers, including 18 cohorts, were included. Reasons for exclusion are presented in the Preferred Reporting Items for Systematic Reviews and Meta-Analyses (PRISMA) flowchart (**Figure 1**). The PRISMA checklist is outlined in **Supplementary Table 2**. An electronic database search identified 19,224 studies; these reduced to 13,197 after removing duplicates. Titles were screened manually for eligibility whereafter 119 articles remained. Full texts were assessed leaving 20 articles. Most common reasons for exclusion were studies assessing the wrong outcome and using non ACB-specific measures. Descriptive information on the included papers can be found in **Table 1**.

**Table 1:** Descriptive data of papers included in review (n = 20), stratified by anticholinergic burden (ACB) measure.

Study	Cohort size (N)	Design	Setting	Country	F/up (months)	Sex (Female)	Age (Mean (SD), unless otherwise specified)
**ACBS**							
Chatterjee et al. (2017)	224,740	Nested case control	Current and former nursing home residents	USA	60 and 90 days prior to death	Cases: 34,819 (77.47%)	Cases: 83.47 (7.64
						Controls: 139,276 (77.47%)	Controls: 83.43 (7.61)
Cross et al. (2017)	964	Retrospective cohort	Patients attending memory clinics	Australia	36 months and 90 days	456 (47.3%)	77.6 (7.4)
Egberts et al. (2017)	905	Retrospective cohort	Geriatric ward admissions	Netherlands	In-hospital mortality (LOS median 8 days, IQR 5-11)	468 (51.7%)	81.0 (7.03)
Fox et al. (2011)	12,423	Prospective cohort	Community-dwelling and institutionalised older persons	England, Wales	24	7,454 (60%)	75 (6.8)
Kidd et al. (2014)	419	Prospective cohort	Acute medical assessment units or acute geriatric ward	England, Scotland	In-hospital mortality – 3 day, 7 day and overall	68%	Median 92.9 (IQR 94.1-95.1)
Lattanzio et al. (2018a)	807	Prospective cohort	Patients discharged from acute geriatric care wards	Italy	12	438 (54.3%)	81 (7.4)
Lattanzio et al. (2018b)	807	Prospective cohort	Patients discharged from acute geriatric care wards	Italy	12	438 (54.3%)	81 (7.4)
Mangoni et al. (2013)	71	Prospective cohort	Patients admitted with hip fractures and scheduled for surgery	Netherlands	3 and 12	70.40%	84 (6)
Vetrano et al. (2016)	3,761	Retrospective cohort	Nursing homes	Italy	60	72%	83 (7)
**ADS**							
Chatterjee et al. (2017)	224,740	Nested case control	Current and former nursing home residents	USA	60 and 90 days prior to death	Cases: 34,819 (77.47%)	Cases: 83.47 (7.64)
						Controls: 139,276 (77.47%)	Controls: 83.43 (7.61)
Mangoni et al. (2013)	71	Prospective cohort	Patients admitted with hip fractures and scheduled for surgery	Netherlands	3 and 12	70.40%	84 (6)
Sarbacker et al. (2017)		Prospective cohort	Hispanic Established Populations for the Epidemiologic Study of the Elderly	USA	108	63.3%	74.56 (95%CI 74.26–74.87)
Sevilla-Sánchez et al. (2018)		Prospective cohort	Acute care geriatric unit	Spain	12	65.50%	86.8 (5.37)
**ARS**							
Aalto et al. (2018)	2432	Prospective cohort	Nursing homes and assisted living facilities	Finland	12	0 DAPs: 886 (74%)	0 DAPs: 85 (7)
						1 DAP: 516 (76%)	1 DAP: 84 (8)
						2 DAPs: 237 (76%)	2 DAPs: 82 (8)
						3+ DAPs: 183 (74%)	3 DAPs: 81 (7)
Egberts et al. (2017)	905	Retrospective cohort	Geriatric ward admissions	Netherlands	In-hospital mortality (LOS median 8 days, IQR 5-11)	468 (51.7%)	81 (7.03)
Gutiérrez-Valencia et al. (2017)	921	Prospective cohort	Patient discharged from geriatric and acute care wards	Italy	12	509 (55.3%)	81.2 (7.4)
Kumpula et al. (2011)	1004	Prospective cohort	Long term care wards (facilities which provide more intensive care than regular nursing homes)	Finland	12	75%	81.3 (10.9)
Landi et al. (2014)	1490	Prospective cohort	Nursing homes	Italy	12	71.50%	ARS 0: 85 (8)
							ARS ≥1: 82 (8)
Lowry et al. (2011)	362	Prospective cohort	Acute geriatric unit	Scotland	In-hospital mortality (LOS median 11 days, IQR 4-24)	59.40%	84 (7)
Mangoni et al. (2013)	71	Prospective cohort	Patients admitted with hip fractures and scheduled for surgery	Netherlands	3 and 12	70.40%	84 (6)
McIsaac et al. (2018)	245,410	Retrospective cohort	Noninstitutionalised patients admitted for an elective, major non-cardiac surgery	Canada	90 days	ARS 0: 49.9%	ARS 0: 74 (6)
						ARS 1-2: 61.0%	ARS 1-2: 74 (6)
						ARS≥3: 60.2%	ARS ≥3: 75 (6)
**DBI _(anticholinergic)_**							
Lowry et al. (2012)	362	Prospective cohort	Acute geriatric unit	Scotland	In-hospital mortality (LOS median 11 days, IQR 4-24)	59.40%	84 (7)
Mangoni et al. (2013)	71	Prospective cohort	Patients admitted with hip fractures and scheduled for surgery	Netherlands	3 and 12	70.40%	84 (6)
**Miscellaneous scales**							
Agar et al. (2010) (Clinician Rated Anti-Cholinergic Scale (modified version) (CrAS))	112	Prospective cohort	Southern Adelaide Palliative Care Services - Inpatients and outpatients	Australia	Until death (8.9 weeks (SD 11.6))	58 (52%)	72 (12)
Egberts et al. (2017) (Chew)	905	Prospective cohort	Geriatric ward admissions	Netherlands	In-hospital mortality (LOS median 8 days, IQR 5-11)	468 (51.7%)	81 (7.03)
Gutiérrez-Valencia et al. (2017) (Duran’s list)	921	Prospective cohort	Patients discharged from acute and geriatric care wards	Italy	12	509 (55.3%)	81.2 (7.4)
Wauters et al. (2017) (MARANTE scale)	503	Prospective cohort	General Practitioner centres	Belgium	18	61%	84.4 (range 80–102)

ACBS, Anticholinergic Cognitive Burden Scale; ADS, Anticholinergic Drug Scale; ARS, Anticholinergic Risk Scale; DAP(s), Drug(s) with anticholinergic properties; DBI (anticholinergic), Anticholinergic Component of the Drug Burden Index; LOS = l, Length of Stay; IQR, Interquartile Range.

A total of 498,056 older individuals participated across the 18 studies, with a sample size range of 71 (Mangoni et al., 2013) to 245,410 (McIsaac et al., 2018) people. Two identical study cohorts were each used twice in separate papers (Lattanzio 2018 A (Lattanzio et al., 2018a) and B (Lattanzio et al., 2018b); Lowry 2011 (Lowry et al., 2011) and 2012 (Lowry et al., 2012)); as a result, participants were only counted once. Ages ranged from mean 71 years (SD 12) (Agar et al., 2010) to median 93 years (IQR 91-95) (Kidd et al., 2014). Fifteen studies were based in Europe, three in North America and two in Australia (see **Table 1**).

Eight ACB measures were used (Han et al., 2001; Carnahan et al., 2006; Hilmer et al., 2007; Boustani et al., 2008; Chew et al., 2008; Rudolph et al., 2008; Durán et al., 2013; Klamer et al., 2017). The most frequently used were the Anticholinergic Cognitive Burden Scale (ACBS; n=9), Anticholinergic Risk Scale (ARS; n=8), Anticholinergic Drug Scale (ADS; n=4) and the Drug Burden Index_(anticholinergic)_ (DBI_(antichol)_; n=2). Measures reported once each were the list of Chew (hereon referred to as “Chew”), Clinician Rated Anticholinergic Scale (CrAS), Duran’s list and the Muscarinic Acetylcholinergic Receptor ANTagonist Exposure (MARANTE) scale. Summaries of each anticholinergic measure can be found in **Supplementary Table 3**. Four papers used more than one ACB measure in their analysis. Sevilla-Sánchez et al. (2018) used two measures, however one of these (DBI_(total)_) was not anticholinergic-specific (see exclusion criteria). The data for this measure were therefore excluded from analysis.

**Figure 2:**
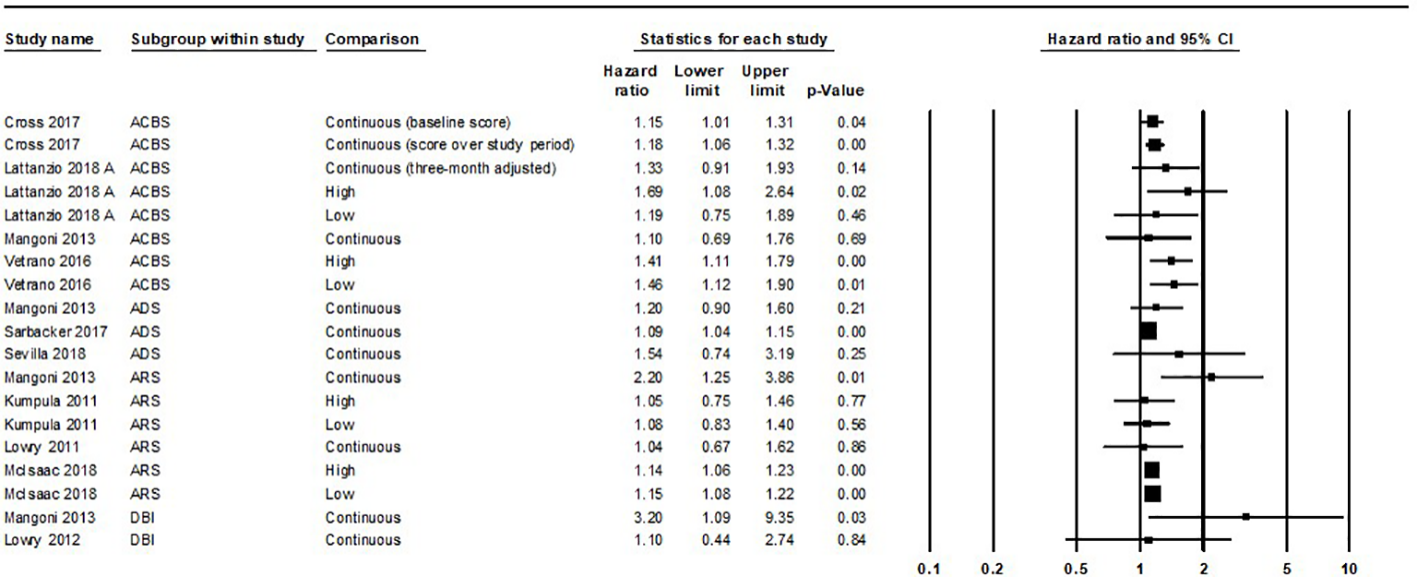
Forest plot of all hazard ratios of all-cause mortality within the evidence base. A summary statistic is not included as the same study population is used more than once. ACBS, Anticholinergic Cognitive Burden Scale; ADS, Anticholinergic Drug Scale; ARS, Anticholinergic Risk Scale; DBI, Anticholinergic Component of the Drug Burden Index. Variation was apparent in how individual studies classed “high” and “low” exposure through ACB measures. Lattanzio 2018 A and Vetrano 2016 classed “low” exposure as a score of ACBS one and “high” exposure as ACBS two or more. Kumpula 2011 and McIsaac 2018 classed “low” exposure as ARS one to two and “high” exposure as ARS three or more.

**Figure 3:**
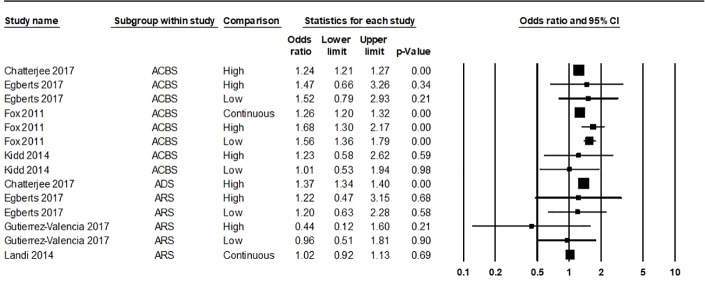
Forest plot of all odds ratios of all-cause mortality within the evidence base. A summary statistic is not included as the same study population is used more than once. ACBS, Anticholinergic Cognitive Burden Scale; ADS, Anticholinergic Drug Scale; ARS, Anticholinergic Risk Scale. Variation was apparent in how individual studies classed “high” and “low” exposure through ACB measures. “Low” anticholinergic exposure data for Chatterjee 2017 was not given. “High” exposure for Chatterjee 2017 ACBS/ADS was classed as a score of 2 or 3. Egberts 2017 classed “low” exposure as ACBS/ARS one to two and “high” exposure as ACBS/ARS three or more. Kidd 2014 classed “low” exposure as ACBS one and “high” exposure as ACBS two or more. Fox 2011 classed “low” exposure as ACBS one or more and “high” as ACBS two or more. Gutierrez-Valencia 2017 classed “low” exposure as ARS one and “high” as ARS two or more.

Forest plot of all hazard ratios and odds ratios of all-cause mortality within the evidence base can be found in **Figures 2** and **3**, respectively. Meaningful meta-analysis of all studies was not possible due to high heterogeneity in study design and reporting.

### Risk of Bias Assessment

**Figure 4:**
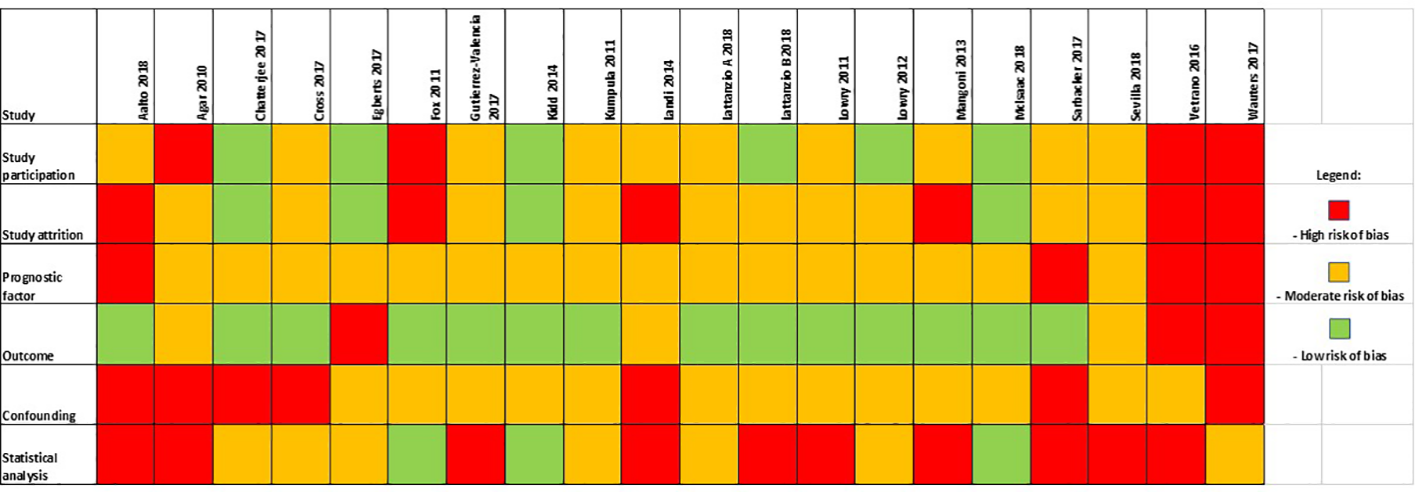
Summarized results of the Quality in Prognosis Studies (QUIPS) risk of bias assessment.

Results of the QUIPS risk of bias assessment are available in **Figure 4**. Of the 20 papers in the final analysis, 15 were considered high risk of bias in at least one or more QUIPS category. Of these, three papers (Vetrano et al., 2016; Wauters et al., 2017; Aalto et al., 2018) had high risk of bias scores in four or more of the six QUIPS categories. A common area of bias included a lack of reporting of prognostic factor measurement—no study had assessed anticholinergic intake by two independent means. Additionally, studies presented with a lack of justification for the adjustment of certain confounders, as well as overall lack of adjustment or possible over-adjustment for confounders. Only two studies adjusted for temporal change in a patient’s ACB exposure. Furthermore, statistical reporting was frequently not detailed and access to study protocols was limited. Frequently, unadjusted results were not reported as opposed to adjusted results, negating the possibility of unadjusted meta-analysis synthesis.

### GRADE Assessment

Full GRADE results can be viewed in **Table 2**. Overall, the quality of the evidence base was considered very low for each ACB measure. Issues arose across the GRADE criteria and included: studies having a high risk of bias, limited generalizability of study setting, small effect sizes, risk of publication bias, and heterogenous reporting of potential dose-effect. For publication bias assessment, it was not possible to build funnel plots due to insufficient studies and variation in statistical effect sizes.

**Table 2:** Results of the Grading of Recommendations, Assessment, Development, and Evaluations (GRADE) analysis.

ACB Measure	No. of participants	No. of studies	No. of cohort	Phase	Study limitations	Inconsistency	Indirectness	Imprecision	Publication Bias	Moderate/large effect size	Dose effect	Overall quality
ACBS	244,090	9	9	Phase 2	Serious^a^	None	Serious^b^	Serious^c^	Serious^d^	Serious^e^	Unclear^f^	Very low
ADS	226,543	4	4	Phase 2	Serious^g^	Serious^h^	Serious^i^	Serious^j^	Serious^d^	Serious^k^	Unclear^l^	Very low
ARS	252,595	8	8	Phase 2	Serious^m^	Serious^n^	Serious^i^	Serious^n^	Serious^d^	Serious^k^	Unclear^l^	Very low
Chew	905	1	1	Phase 2	Serious^o^	Serious^l^	Serious^p^	Serious^d^	Serious^d^	Serious^k^	Unclear^l^	Very low
CRAS	112	1	1	Phase 2	Very Serious^q^	Serious^l^	Serious^r^	Serious^l^	Serious^l^	Unclear^l^	Unclear^l^	Very low
Duran’s List	921	1	1	Phase 2	Serious^s^	Serious^l^	Serious^p^	Serious^d^	Serious^d^	Serious^k^	Unclear^l^	Very low
DBI ^(antichol)^	433	2	2	Phase 2	Serious^t^	Serious^u^	Serious^p^	Serious^v^	Serious^d^	Serious^w^	Unclear^l^	Very low
MARANTE Scale	503	1	1	Phase 2	Very Serious^x^	Serious^l^	None	Serious^y^	Serious^d^	Serious^k^	Unclear^d^	Very low

Grade assessment with justification given as follows: ^a^Risk of bias (ROB): 7/9 studies are at high ROB in ≥1 Quality in Prognosis Studies (QUIPS) category, all studies have moderate ROB. Common issues surrounding prognostic factor use and poor statistical use/reporting. ^b^Studies conducted in range of unique settings (acute care, nursing homes, community). ^c^>50% of CIs included no effect. ^d^Too few studies. ^e^Of the 9 studies, OR were small (95%CI 1.01–1.68) and HR were small (1.09–1.69). ^f^Unclear—not all studies were consistent in showing dose effect. ^g^Risk of bias (ROB): All studies are at high ROB in ≥1 QUIPS category, all are at moderate ROB in ≥2 QUIPS categories. Common issues surrounding prognostic factor measurement and poor statistical use/reporting. ^h^CIs of 3/4 studies included no effect. ^i^Studies conducted in range of unique settings (acute care, nursing homes). ^j^>75% of CIs included no effect. ^k^All effect sizes small. ^l^Not possible to determine. ^m^Risk of bias (ROB): 6/8 studies are high ROB in ≥1 QUIPS category, all studies are moderate in ≥1 QUIPS category. Common issues surrounding attrition, confounding and poor statistical analysis. ^n^CIs of 5/8 studies included no effect. ^o^Risk of bias (ROB): High ROB in 1 QUIPS category, moderate ROB in 3 QUIPS categories, rate of outcome too low and poor reporting. ^p^Restricted to acute care patients only. ^q^Risk of bias (ROB): High ROB in 3 QUIPS categories, moderate ROB in 3 QUIPS categories. ^r^Restricted to palliative care. ^s^Risk of bias (ROB): High ROB in 1 QUIPS category, moderate ROB in 4 QUIPS categories, statistical analysis poorly described. ^t^Risk of bias (ROB): High ROB in ≥1 QUIPS category for both studies, common issues surrounding attrition and poor reporting of statistics. ^u^CIs for 1/2 studies include no effect, one study is unadjusted. ^v^Large CI for one study (95%CI 1.09-9.35), this data is also unadjusted. ^w^One small and one moderate effect size but inconsistent significance and one large CI. ^x^Risk of bias (ROB): 5/6 QUIPS categories are at high ROB. ^y^CIs include no effect.

### Anticholinergic Cognitive Burden Scale (ACBS)

Full information regarding study results can be found in **Table 3**. Nine studies Chatterjee et al. (2017), Cross et al. (2017), Egberts et al. (2017), Fox et al. (2011), Kidd et al. (2014), Lattanzio et al., (2018a), Lattanzio et al. (2018b), Mangoni et al. (2013) and Vetrano et al. (2016) involving 244,090 individuals were included, with cohort sizes ranging from 71 Mangoni et al. (2013) to 224,740 Chatterjee et al. (2017). Two studies Lattanzio et al. (2018a); Lattanzio et al. (2018b) utilized the same study population; subsequently, their population was only counted once within the total. Five took place in hospital settings, two in nursing homes, one incorporated community and nursing home residents and one in an outpatient clinic setting.

**Table 3:** Summary of results of papers included in review (n=20), stratified by anticholinergic burden (ACB) measure.

Study	Cohort size (N)	Design	Setting	F/up (months)	Unadjusted Results – HR/OR (95%CI)	Adjusted Results – HR/OR (95%CI)
**ACBS**						
Chatterjee et al. (2017)	224,740	Nested case control	Current and former nursing home residents	60 and 90 days prior to death	NR	ACBS level 2/3, prescribed within 90 days: OR 1.24 (1.21, 1.27)^a^
Cross et al. (2017)	964	Retrospective cohort	Patients attending memory clinics	36 months and 90 days	Baseline ACBS score HR 1.27 (1.15-1.40)	Baseline ACBS score: HR 1.15 (1.01-1.31)^b^
					ACBS score over study period HR 1.29 (1.19-1.39)	ACBS score over study period: HR 1.18 (1.06-1.32)^b^
Egberts et al. (2017)	905	Retrospective cohort	Geriatric ward admissions	Up to discharge (median 8 days, IQR 5-11)	NR	Low (1-2): OR 1.52 (0.79–2.93)^c^
						High (≥3): OR 1.47 (0.66–3.25)^c^
Fox et al. (2011)	12,423	Prospective cohort	Community-dwelling and institutionalised older persons	24	NR	Continuous: OR 1.26 (1.20–1.32)^d^
						Low (≥1): OR 1.56 (1.36–1.79)^d^
						High (≥2): OR 1.68 (1.30–2.16)^d^
Kidd et al. (2014)	419	Prospective cohort	Acute medical assessment units or acute geriatric ward	In-hospital mortality – 3 day, 7 day and overall	Overall mortality: 1: OR 1.04 (0.58–1.86)	Overall mortality: Low (1): OR 1.01 (0.53–1.95)^f^
					≥2: OR 1.10 (0.60–2.04)	High (≥2): OR 1.23 (0.58–2.63)^f^
Lattanzio et al. (2018a)	807	Prospective cohort	Patients discharged from acute geriatric care wards	12	NR	Low (1): HR 1.19 (0.75–1.90)^i^
						High (≥2): HR 1.69 (1.09–2.65)^i^
						Adjusted for ACBS at 3-month follow-up: HR 1.33 (0.97–2.05)^i^
Lattanzio et al. (2018b)	807	Prospective cohort	Patients discharged from acute geriatric care wards	12	NR	No BADL dependency (n = 537) Low (1): HR 0.92 (0.45–1.89)^j^ High (≥2): HR 1.06 (0.50–2.34)^j^ Continuous: HR 0.98 (0.75–1.28)^j^
						Dependency in ≥ 1 BADL (n= 270) Low (1): HR 1.50 (0.81-2.73)^j^ High (≥2): HR 2.25 (1.22–4.14)^j^ Continuous: HR 1.28 (1.11–1.49)^j^
						Adjusted for ACBS at 3-month follow-up (with BADL dependency) High (≥2): HR 2.18 (1.20–3.98)^j^
Mangoni et al. (2013)	71	Prospective cohort	Patients admitted with hip fractures and scheduled for surgery	3 and 12	Univariate analysis of 3-month mortality Continuous: HR 1.1 (0.7–1.8)	NR
					Univariate analysis of 1-year mortality Continuous: HR 1.1 (0.7–1.8)	NR
Mangoni et al. (2013)	3,761	Retrospective cohort	Nursing homes	60	NR	Low (1): HR 1.46 (1.12–1.9)^p^
						High (≥2): HR 1.41 (1.11–1.79)^p^
**ADS**						
Chatterjee et al. (2017)	224,740	Nested case control	Current and former nursing home residents	60 and 90 days prior to death		ADS level 2/3 (high), prescribed within 90 days: OR 1.37(1.34, 1.40)^a^
Mangoni et al. (2013)	71	Prospective cohort	Patients admitted with hip fractures and scheduled for surgery	3 and 12	Univariate analysis of 3-month mortality HR 1.3 (0.9–1.9)	NR
					Univariate analysis of 1-year mortality HR 1.2 (0.9–1.6)	
Sarbacker et al. (2017)	1,497	Prospective cohort	Hispanic Established Populations for the Epidemiologic Study of the Elderly	108 (9 years)	HR 1.12 (1.07–1.17)	Continuous: HR 1.09 (1.04–1.15)^n^
Sevilla-Sánchez et al. (2018)	235	Prospective cohort	Acute care geriatric unit	12	NR	Continuous: HR 1.54 (0.74–3.18)^o^
						High (> 3) vs low (< 3) use: HR 1.05 (0.75–1.47)^o^
**ARS**						
Aalto et al. (2018)	2,432	Prospective cohort	Nursing homes and assissted living facilties	12	No. of deaths during 1 year follow up (%) p value: 0.11	NR
					0 DAPs: 238 (20%)	
					1 DAP: 134 (20%)	
					2 DAPs: 56 (18%)	
					3+ DAPs: 50 (20%)	
Egberts et al. (2017)	905	Retrospective cohort	Geriatric ward admissions	Up to discharge (median 8 days, 5–11 IQR)	NR	Low (1-2): OR 1.2 (0.63–2.27)^c^
						High (≥3): OR 1.22 (0.47–3.13)^c^
Gutiérrez-Valencia et al. (2017)	921	Prospective cohort	Patient discharged from geriatric and acute care wards	12	1: 1.68 (1.10, 2.57)	Low (1): 0.96 (0.51, 1.81)^e^
					≥2: 0.87 (0.4, 1.91)	High (≥2): 0.44 (0.12, 1.59)^e^
Kumpula et al. (2011)	1,004	Prospective cohort	Long term care wards	12	NR	Low (1-2): HR 1.08 (0.84–1.41)^g^
						High (≥3): HR 1.05 (0.75–1.46)^g^
Landi et al. (2014)	1,490	Prospective cohort	Nursing homes	6 and 12		Continuous: OR 1.02 (0.95–1.17)^h^
Lowry et al. (2011)	362	Prospective cohort	Acute geriatric unit	In-hospital mortality (LOS median 11 days, IQR 4-24)	Continuous: HR 1.31 (0.97–1.77)	Continuous: 1.04 (0.67–1.62)^k^
					ARS (dose- adjusted) continuous: 1.43 (0.97-2.12)	ARS (dose- adjusted) continuous: 1.12 (0.64–1.96)^k^
Mangoni et al. (2013)	71	Prospective cohort	Patients admitted with hip fractures and scheduled for surgery	3 and 12	Univariate analysis of 3-month mortality Continuous: HR 1.6 (1.2–2.2)	Multivariate 3-month mortality Continuous: HR 2.2 (1.2-3.7)^l^
					Univariate analysis of 1-year mortality Continuous: HR 1.4 (1.1–1.8)	
McIsaac et al. (2018)	245,410	Retrospective cohort	Noninstitutionalised patients admitted for an elective, major non-cardiac surgery	90 days	1-2: HR 1.49 (1.40–1.59)	Low (1-2): HR 1.15 (1.08–1.22)^m^
					≥3: HR 1.39 (1.30–1.49)	High (≥3): HR 1.14 (1.06–1.23)^m^
**DBI ^(anticholinergic)^**						
Lowry et al. (2012)	362	Prospective cohort	Acute geriatric unit	In-hospital mortality (LOS median 11 days, IQR 4-24)	Continuous: HR 1.09 (0.46–2.57)	Continuous: HR 1.10 (0.44–2.74)^k^
Mangoni et al. (2013)	71	Prospective cohort	Patients admitted with hip fractures and scheduled for surgery	3 and 12	Univariate analysis of 3-month mortality Continuous: HR 4.5 (1.2–16.7)	NR
					Univariate analysis of 1-year mortality Continuous: HR 3.2 (1.1–9.4)	
**Miscellaneous scales**						
Agar et al. (2010) [Clinician Rated Anti-Cholinergic Scale (modified version)]	112	Prospective cohort	Southern Adelaide Palliative Care Services - Inpatients and outpatients	Until death (mean survival time: 8.9 weeks (SD 11.6, median 5.3, IQR 0.2-84.4)	“Log-rank data showed no evidence that survival differed significantly between the three groups”	
Egberts et al. (2017) (Chew)	905	Prospective cohort	Geriatric ward admissions	Until hospital discharge (LOS median 8 days, IQR 5-11)	NR	Low (0.5–1): OR 1.01 (0.56–1.83)^c^
						High (≥1.5): OR 1.39 (0.66–2.92)^c^
Gutiérrez-Valencia et al. (2017) (Duran’s list)	921	Prospective cohort	Patients discharged from acute and geriatric care wards	12	1: OR 1.84 (1.27, 2.65)	Low (1): OR 1.69 (1.02, 2.82)^e^
					≥2: OR 1.52 (0.86, 2.68)	High (≥2): OR 1.52 (0.86, 2.68)^e^
Wauters et al. (2017) (MARANTE scale)	503	Prospective cohort	General Practitioner centres	18	Continuous: HR 1.22 (1.02–1.47)	Continuous: HR 1.09 (0.87–1.36)^q^
					Low (0.5–1.5): HR 1.52 (0.68–3.39)	Low (0.5–1.5): HR 1.31 (0.57–3.02)^q^
					High (≥2): HR 2.77 (1.43–5.38)	High (≥2): HR 2.20 (1.03–4.67)^q^

Papers appear multiple times throughout this table if they have reported their results using more than one ACB measure. “Continuous” denotes when an ACB measure has been used as a continuous variable during analysis. ACBS, Anticholinergic Cognitive Burden Scale; ADS, Anticholinergic Drug Scale; ARS, Anticholinergic Risk Scale; BADL, Basic Activities of Daily Living; DAP(s), Drug(s) with anticholinergic properties; DBI (anticholinergic), anticholinergic component of the Drug Burden Index; HR, Hazard Ratio; IQR, Interquartile Range; LOS, Length of Stay; NR, Not reported; OR, Odds Ratio. Adjustments: ^a^Adjusted for demographic characteristics such as race; co-morbidities such as myocardial infarction, heart failure, vascular diseases, dementia, cerebrovascular events, rheumatological diseases, mild liver disease, pulmonary disorders, renal diseases, ulcer, hemiplegia, diabetes, cancer, metastasis, moderate/chronic liver disease; and duration of depression. ^b^Baseline age, gender, education, dementia/MCI diagnosis, total number of medications, MDBI score, MMSE, SMAF, and NPI score. ^c^Age, sex, Charlson Comorbidity Index (CCI), number of nonanticholinergic drugs and delirium at any time during the hospital stay. ^d^Age, sex, baseline MMSE score, education, social class, number of nonanticholinergic medications, and number of health conditions. ^e^Age, sex, number of chronic diseases, MMSE, impaired ADL and number of non-anticholinergic medications. ^f^Age, no of pre-morbid conditions, ischemic heart disease, no of medications, creatinine and urea. ^g^Adjusted for age, sex, mini nutritional assessment. ^h^Age, gender, comorbidities, baseline functional impairment and cognitive impairment. ^i^Age, sex, cognitive impairment, depression, number of lost BADL, hypertension, heart failure, diabetes, coronary artery disease, atrial ﬁbrillation, peripheral arterial disease, stroke, chronic obstructive pulmonary disease, cancer and number of medications. ^j^Age, sex, cognitive impairment, history of falls, depression, number of medications and hypertension, heart failure, diabetes, coronary artery disease, atrial ﬁbrillation, peripheral arterial disease, stroke, chronic obstructive pulmonary disease, and cancer. ^k^Adjusted for age, sex, institution, dementia, CCI, number of nonanticholinergic drugs, hospital site, Barthel Index category (under 50 vs. 50+). ^l^Age, sex, CCI and preadmission cognitive impairment. ^m^Age, sex, income quartile, comorbidities, one-year mortality risk, total hip replacement (please see paper for extensive list of covariates). ^n^Age, sex, any self-reported diabetes, stroke, smoking, hypertension and cancer. ^o^Age, sex, provenance, age-adjusted CCI, cognitive impairment and geriatric syndromes. ^p^Age, sex, activities of daily living, Depression Rating Scale, Cognitive Performance Scale, dementia, heart failure, stroke, chronic obstructive pulmonary disease, cancer, diabetes, IHD, and hip fracture. ^q^Number of medications & level of multi-morbidity (0–9).

**Figure 5:**
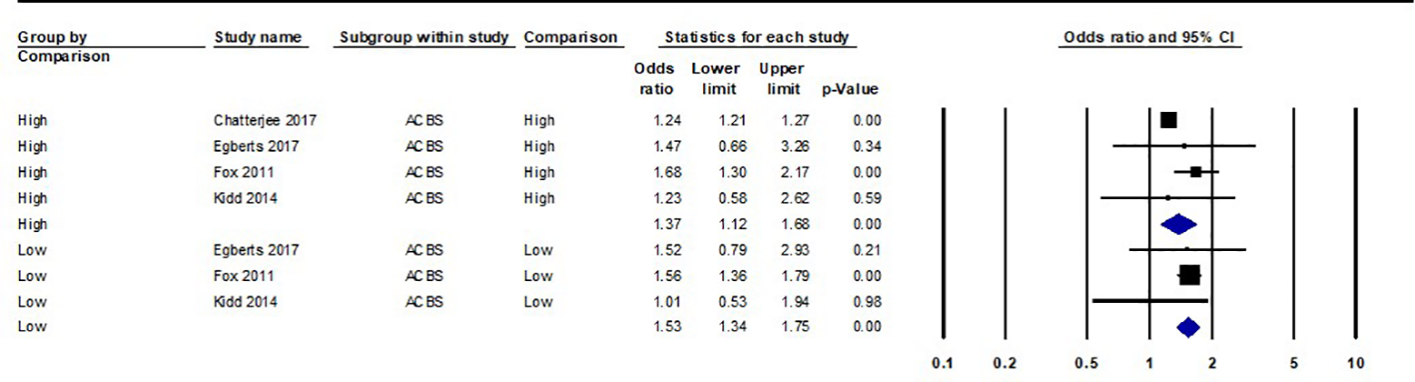
Forest plot of odds ratios of all-cause mortality grouped by anticholinergic exposure level using the ACBS (Anticholinergic Cognitive Burden Scale). “Low” anticholinergic exposure data for Chatterjee 2017 was not given. “High” exposure for Chatterjee 2017 ACBS was classed as a score of 2 or 3. Egberts 2017 classed “low” exposure as ACBS one to two and “high” exposure as ACBS three or more. Kidd 2014 classed “low” exposure as ACBS one and “high” exposure as ACBS two or more. Fox 2011 classed “low” exposure as ACBS one or more and “high” as ACBS two or more.

Summary statistics for “low” anticholinergic exposure measured with the ACBS were HR 1.39 (95%CI 1.1–1.75) and OR 1.53 (95%CI 1.34–1.75); for “high” ACBS these were HR 1.47 (95%CI 1.19–1.81) and OR 1.37 (95% 1.12–1.68) **Figure 5**. Continuous data was analyzed separately, yielding a summary HR of 1.87 (95%CI 1.07–1.32), however it should be noted that within this, results from Mangoni et al. (2013) were unadjusted.

Data for Lattanzio et al. (2018b) was not included in meta-analysis so as not to include the same cohort twice. However, the results from this study were stratified into individuals with a dependency in one or more Basic Activities of Daily Living (BADL) and those without. These results indicated that those who were dependent in at least one BADL were more likely to have a significant association between greater ACB and mortality (HR 1.28 (95%CI 1.11–1.49)) than those who did not (HR 0.98 (95%CI 0.75–1.28). Results from Mangoni et al. (2013) were unadjusted.

### Anticholinergic Drug Scale (ADS)

The ADS was reported in four papers Chatterjee et al. (2017), Mangoni et al. (2013), Sarbacker et al. (2017), and Sevilla-Sánchez et al. (2018). The total population size was 226,543 individuals, with sample sizes ranging from 71 Mangoni et al. (2013) to 224,740 Chatterjee et al. (2017). Two studies took place in hospital settings, one in nursing homes and one was community-based.

Meta-analysis of three studies Mangoni et al. (2013), Sarbacker 2017 (Sarbacker et al., 2017), Sevilla-Sánchez et al. (2018) produced a summary HR of 1.1 (95%CI 1.04–1.15). Data not included in the meta-analysis Chatterjee et al. (2017), comparing “high” ADS (ADS 2/3) to no ADS 90 days before death indicated a stronger association [OR 1.37 (95%CI 1.34–1.4) (**Figure 2**)]. Data for low or continuous ACB within the Chatterjee et al. (2017) cohort were not reported. Results from Mangoni et al. (2013) were unadjusted.

### Anticholinergic Risk Scale (ARS)

Eight papers reported use of the ARS Aalto et al. (2018), Egberts et al. (2017), Gutiérrez-Valencia et al. (2017), Kumpula et al. (2011), Landi et al. (2014), Lowry et al. (2011), Mangoni et al. (2013), McIsaac et al. (2018). Cohort sizes ranged from 71 Mangoni et al. (2013) to 245,410 McIsaac et al. (2018) and included 252,595 participants. Six cohorts were based in hospital settings and two were in nursing home settings.

Meta-analysis of categorical data for “low” ARS produced summary HR 1.15 (95%CI 1.08–1.22) and OR 1.07 (95%CI 0.68–1.68) and “high” ARS produced HR 1.14 (95%CI 1.06–1.22) and OR 0.81 (95%CI 0.3–2.16). A further two studies Lowry et al. (2011), Mangoni et al. (2013) comprised a meta-analysis of continuous ARS data and resulted in a summary HR of 1.48 (95%CI 0.71–3.08).

Two papers were not included in the meta-analysis Aalto et al. (2018), Landi et al. (2014). It should be noted that the cumulative nature of the ARS measure was not applied in the analysis of Aalto et al. (2018); rather, the medications listed within the ARS were used to sum the number of anticholinergic medications a patient was taking. Landi et al. (2014) reported OR 1.02 (95%CI 1.02–0.92–1.12).

### Drug Burden Index—Anticholinergic Component (DBI_(antichol)_)

Two prospective cohort studies reported use of the DBI_(antichol)_ [Lowry 2012 (Agar et al., 2010), Mangoni et al. (2013) and included a total population of 433 older patients in acute care settings. Lowry 2012 (Agar et al., 2010) reported HR 1.10 (95%CI 0.44–2.74) and Mangoni et al. (2013) reported HR 3.2 (1.1–9.4). Results from Mangoni et al. (2013) [HR 3.2 (95%CI 1.1–9.35)] were unadjusted.

### Other ACB Measures

Four papers (three prospective cohorts, one retrospective cohort) reported measures used only once each Agar et al. (2010), Egberts et al. (2017), Gutiérrez-Valencia et al. (2017), Wauters et al. (2017). These measures were: Clinician Rated Anticholinergic Scale (CrAS); Chew; Duran’s list; MARANTE.

Agar et al. (2010) stated “Log-rank data showed no evidence that survival differed significantly between the three groups,” however, no quantitative data were provided. Significant associations between ACB and mortality were produced by Gutiérrez-Valencia et al. (2017) [Duran’s=1, OR 1.69 (95%CI 1.02–2.82) and Wauters et al. (2017) (MARANTE “high” ≥2: HR 2.20 (95%CI 1.03–4.67)].

### A Comparison of ACB Measures

Four papers utilized more than one measure to assess ACB within the same cohort of individuals Chatterjee et al. (2017), Egberts et al. (2017), Gutiérrez-Valencia et al. (2017), Mangoni et al. (2013). Chatterjee et al. (2017) compared ADS with ACBS, whereby ADS resulted in significantly higher odds ratios than ACBS [ADS level 2/3: OR 1.37 (95%CI 1.34, 1.40); ACBS level 2/3: OR 1.24 (95%CI 1.21, 1.27)]. No data were given for low anticholinergic exposure for either scale.

Data provided by Egberts et al. (2017) compared ARS, ACBS, and Chew. Most results showed no significant association, with wide CIs (**Table 3**). ACBS exhibited higher odds ratios compared to ARS and Chew, but the differences were not statistically significant. Gutiérrez-Valencia et al. (2017) compared ARS and Duran’s list. There was a positive association between ACB measured by Duran’s list and mortality (OR 1.69 (95%CI 1.02–2.82), while the association between ARS and mortality was not significant. Mangoni et al. (2013) compared ACBS, ADS, ARS, and DBI_(antichol)_, however, interpretation of these results were limited as analyses for all measures (apart from ARS three-month mortality) were not adjusted for confounders. Hazard ratios were small and not statistically significant for ACBS and ADS and increased with ARS (HR 2.2 (95%CI 1.2–3.7) and DBI_(antichol)_ (HR 3.2 (95%CI 1.1–9.4)).

## Discussion

This is the first systematic review and meta-analysis assessing the prognostic utility of individual ACB measures to predict mortality specifically in individuals ≥65 years. Of 20 included studies, 11 reported a significant association between ACB and mortality and meta-analysis showed a positive association between greater ACB and mortality. However, a dose-effect response was not consistently apparent. The most frequently used ACB measures were ACBS, ARS and ADS. Inter-study variation and methodological heterogeneity between studies precluded combined meta-analyses across all scales.

Our finding that greater ACB appears associated with increased risk of mortality conflicts with the findings reported in the Fox et al. (2014) systematic review, where no significant association between ACB and mortality was identified. This may be because our review focused on older people, where risk of mortality is much higher. Also, the current review focused on use of any published ACB scale whilst their review relied on defining ACB where medications were included in the ACBS, a scale developed to predict cognitive decline rather than mortality. Another explanation may be that the majority (n=14) of our included studies were published post-2014, in line with increasing interest in this topic area, providing a larger pool of data to analyze. Specifically, this allowed the production of summary statistics, which were not possible in the Fox et al. (2014) review.

It was not possible for us to declare one scale as superior to another. Larger odds and hazard ratios were seen with the ACBS, supporting the findings of Ruxton et al. (2015) where the ACBS showed the largest effect sizes. The discrepancies in effect sizes between scales may be a result of the variations in how differing ACB measures assess the potency of an anticholinergic agent, as highlighted by Salahudeen et al. (2015). For example, the ACBS was developed through systematic review and a multi-disciplinary panel to identify anticholinergic medications and assess their potency (Boustani et al., 2008). On the other hand, the ARS was developed by geriatricians and pharmacists within the Veterans Affairs Boston Healthcare System *via* a literature review of the most commonly prescribed medications among predominantly male, veteran patients (Rudolph et al., 2008). The varying number of drugs with anticholinergic properties included in each measure, along with their graded potencies, may contribute towards variations in risk seen in the current review. The fact the three most frequently used measures (ACBS, ARS, ADS) were all developed in the USA may limit applications internationally due to differing drug formularies between countries.

While our meta-analysis adds helpful new information to the existing literature base, there are limitations that should be noted. Firstly, the reporting of results between studies varied greatly; this led to multiple levels of stratification before data could be grouped for meta-analysis. Consequently, a large combined meta-analysis, including all 20 studies, could not be created and meaningfully interpreted. Once stratified by ACB measure, data were limited, with some single meta-analyses containing only two studies. Additionally, many studies did not have long enough follow-up times. Also, the DBI_(antichol)_ is the only ACB measure which takes into account the dose of drug being administered to a patient, yet it was only used twice in the current review, limiting the ability to interpret whether having a scale which takes dose into account is of benefit in assessing morality risk. Finally, the indication of anticholinergic medications in each study is not known. It is possible this may have an impact on mortality risk; while those being treated for multiple conditions are more likely to be users of anticholinergic medications, multi-morbidity alone is associated with mortality risk (Wauters et al., 2017).

A clear advantage of the current study was the use of contemporary best practice guidance for prognostic reviews; the framework by the Cochrane Prognostic Review Group was used in order to ensure the methodology and reporting within this review adhered to high quality standards.

Another advantage of the current study was the analysis of dose-effect within ACB measures (where possible), something not apparent within other systematic reviews. In theory, a clear increase in odds should have been visible in “high” exposure groups compared to “low” throughout scales. Not only was this not visible in odds data for ACBS and ARS when grouped by exposure level, but the inverse was apparent on these two occasions. A contributing factor may have been the tendency for participant numbers to be far lower in “high” exposure categories, yet other clear mechanisms for this have not yet been established. Further advantages were that this study assessed the risk of bias using an appropriate prognostic review risk of bias tool, QUIPS, and the body of evidence was assessed using the GRADE tool (adapted specifically for prognostic studies). This provided a clear understanding of the quality of the current evidence and where improvements in future research can be made. Specifically, there is a need for large-scale cohort studies supported by adequate sample size calculations based on clinically meaningful effect sizes. The use of multiple ACB measures on the same population reduces risk from heterogeneity and would increase confidence in making comparisons across the various ACB measures. Next, if a follow-up period of over one year is used, then repeated ACB measurements would prove useful to control for temporal changes in patients’ anticholinergic exposure. Most studies within the review did not adjust for temporal changes in ACB, yet it is evident that patients’ prescriptions, and therefore ACB, will change over time (Lu et al., 2015), which may have influenced risk.

Our findings provide further evidence of the risks associated with the use of anticholinergic medications. This will help with clinical decision making. Being aware of, and able to discuss available evidence with patients will allow clinicians to make more informed treatment decisions. From a provider perspective, the economic implications of the use of anticholinergic medications are not known and warrant further investigation. At present we do not know how reducing or stopping anticholinergic medications, and/or switching to non-anticholinergic alternatives, may impact upon the costs of patient care. We recommend those designing future trials aiming to de-prescribe anticholinergic medications build economic evaluation into their design.

## Conclusion

This systematic review identified twenty studies assessing ACB and mortality using anticholinergic-specific measures in older adults. Meta-analysis indicated an association between anticholinergic exposure and higher risk of mortality using the ACBS, ARS, and ADS. The evidence base was of poor quality overall, with moderate to high risk of bias such that it was not possible to ascertain if any ACB measure was superior to another, though the ACBS generally exhibited larger effect sizes than other ACB measures. Future large-scale high-quality research investigating validated measures is recommended. Meanwhile, this review’s findings further add to the evidence base that clinicians should be cautious around prescribing anticholinergic medications to older persons.

## Data Availability Statement

All datasets generated for this study are included in the article/**Supplementary Material**.

## Author Contributions

Wrote manuscript: KG-M, CS, RS, MT-R, TQ, YL, and PM. Designed research: CS, RS, TQ, YL, and PM. Performed research: KG-M, CS, RS, MT-R, TQ, and PM. Analyzed data: KG-M, CS, RS, MT-R, TQ, and YL.

## Funding

This work was supported by The Dunhill Medical Trust (grant number RPGF1806/66) and KG-M received an Aberdeen Summer Research Scholarship funded by the Institute of Applied Health Sciences as part of Aberdeen Clinical Academic Training (ACAT) Programme.

## Conflict of Interest

The authors declare that the research was conducted in the absence of any commercial or financial relationships that could be construed as a potential conflict of interest.
