# Supplementary material for: The Prognostic Value of Anticholinergic Burden Measures in Relation to Mortality in Older Individuals: A Systematic Review and Meta-Analysis
Source: Front Pharmacol. 2020 Apr 29;11:570. doi: 10.3389/fphar.2020.00570 (PMC7201087; doi:10.3389/fphar.2020.00570)
Supplement: Supplementary file 1 [file DataSheet_1.docx]

Supplementary Material

## **Supplementary tables**

**Supplementary Table 1:** Database search strategy.

|  | **Ovid Medline** | | **EMBASE** | | **CINAHL** | | **PsycInfo** | |
| --- | --- | --- | --- | --- | --- | --- | --- | --- |
| **ACH** | (MH cholinergic agents or cholinergic antagonists or muscarinic antagonists or nicotinic antagonists) OR  (cholinergic* or anti-cholinergic* or anticholinergic* or chews list or summers list or elletts list or muscarinic).tw. OR  (cholinergic* or anti-cholinergic* or anticholinergic* or chews list or summers list or elletts list or muscarinic).kw. | **74553** | (MH cholinergic receptor blocking agent or cholinergic receptor affecting agent or cholinergic receptor stimulating agent) OR (cholinergic* or anti-cholinergic* or anticholinergic* or chews list or summers list or elletts list or muscarinic).tw. OR  cholinergic* or anti-cholinergic* or anticholinergic* or chews list or summers list or elletts list or muscarinic).kw. | **109821** | (MM cholinergic antagonists or cholinergic agents or cholinergic agonists or nicotinic agonists or muscarinic agonists ) OR (cholinergic* or anti-cholinergic* or anticholinergic* or chews list or summers list or elletts list or muscarinic).tx. | **6440** | (MH cholinergic drugs or cholinergic blocking drugs) OR (cholinergic* or anti-cholinergic* or anticholinergic* or chews list or summers list or elletts list or muscarinic).tw. OR  cholinergic* or anti-cholinergic* or anticholinergic* or chews list or summers list or elletts list or muscarinic).id. | **15250** |
| **Prognostic** | Exp. predictive value of tests or observer variation  OR  Predict*.ti or (valid* or rule*).af.  OR  Predict*.ti and (outcome* or risk* or model*)  OR  ((history* or variable* or criteria or scor* or characteristic* or finding* or factor*) and (predict* or model* or decision* or identif* or prognos*)).af.  OR  (decision* and (model* or clinical* or logistic models*)).af.  OR  (prognostic* and( history or variable* or criteria or scor* or characteristic* or finding* or factor* or model*)).af. | **4222923** | Exp. predictive value or observer variation or predicator variable  OR  Predict*.ti or (valid* or rule*).af.  OR  Predict*.ti and (outcome* or risk* or model*)  OR  ((history* or variable* or criteria or scor* or characteristic* or finding* or factor*) and (predict* or model* or decision* or identif* or prognos*)).af.  OR  (decision* and (model* or clinical* or logistic models*)).af.  OR  (prognostic* and( history or variable* or criteria or scor* or characteristic* or finding* or factor* or model*)).af. | **4901495** | Exp. predictive value of tests  OR  Predict*.tx or (valid* or rule*).tx.  OR  Predict*.tx and (outcome* or risk* or model*)  OR  ((history* or variable* or criteria or scor* or characteristic* or finding* or factor*) and (predict* or model* or decision* or identif* or prognos*)).tx.  OR  (decision* and (model* or clinical* or logistic models*)).tx.  OR  (prognostic* and( history or variable* or criteria or scor* or characteristic* or finding* or factor* or model*)).tx. | **853653** | Exp. prediction or predictability or prognosis or interrater reliability  OR  Predict*.ti or (valid* or rule*).af.  OR  Predict*.ti and (outcome* or risk* or model*)  OR  ((history* or variable* or criteria or scor* or characteristic* or finding* or factor*) and (predict* or model* or decision* or identif* or prognos*)).af.  OR  (decision* and (model* or clinical* or logistic models*)).af.  OR  (prognostic* and( history or variable* or criteria or scor* or characteristic* or finding* or factor* or model*)).af. | **2081463** |
|  | **ACB & Prognostics**  **Limit: 2006-present** | **8438**  **4759** | **ACB & Prognostics**  **Limit: 2006-present** | **12164**  **8036** | **ACB & Prognostics**  **Limit: 2006-present** | **1190**  **1047** | **ACB & Prognostics**  **Limit: 2006-present** | **6197**  **4430** |
| **Scale** | Anticholinergic effect on cognition scale or anticholinergic impregnation scale or anticholinergic drug scale or anticholinergic activity scale or clinician rated anticholinergic scale or muscarinic acetylcholinergic receptor antagonist scale or anticholinergic risk scale or anticholinergic loading scale or anticholinergic cognitive burden scale or anticholinergic burden classification or modified anticholinergic risk scale or serum anticholinergic activity or drug burden index or chews list or summers list or elletts list.tw.  Limit: 2006-Present | **228** | Anticholinergic effect on cognition scale or anticholinergic impregnation scale or anticholinergic drug scale or anticholinergic activity scale or clinician rated anticholinergic scale or muscarinic acetylcholinergic receptor antagonist scale or anticholinergic risk scale or anticholinergic loading scale or anticholinergic cognitive burden scale or anticholinergic burden classification or modified anticholinergic risk scale or serum anticholinergic activity or drug burden index or chews list or summers list or elletts list.tw.  Limit: 2006-Present | **389** | Anticholinergic effect on cognition scale or anticholinergic impregnation scale or anticholinergic drug scale or anticholinergic activity scale or clinician rated anticholinergic scale or muscarinic acetylcholinergic receptor antagonist scale or anticholinergic risk scale or anticholinergic loading scale or anticholinergic cognitive burden scale or anticholinergic burden classification or modified anticholinergic risk scale or serum anticholinergic activity or drug burden index or chews list or summers list or elletts list.tw.  Limit: 2006-Present | **195** | Anticholinergic effect on cognition scale or anticholinergic impregnation scale or anticholinergic drug scale or anticholinergic activity scale or clinician rated anticholinergic scale or muscarinic acetylcholinergic receptor antagonist scale or anticholinergic risk scale or anticholinergic loading scale or anticholinergic cognitive burden scale or anticholinergic burden classification or modified anticholinergic risk scale or serum anticholinergic activity or drug burden index or chews list or summers list or elletts list.tw.  Limit: 2006-Present | **75** |
|  | (ACB & Prognostics) or Scale.tw.  Limit:2006- present | **4987** | (ACB & Prognostics) or Scale.tw.  Limit:2006- present | **8499** | (ACB & Prognostics) or Scale.tw.  Limit:2006- present | **1241** | (ACB & Prognostics) or Scale.tw.  Limit:2006- present | **4517** |

**Supplementary Table 2:** PRISMA checklist.

| **Section/topic** | **#** | **Checklist item** | **Reported on page #** |
| --- | --- | --- | --- |
| **TITLE** | | |  |
| Title | 1 | Identify the report as a systematic review, meta-analysis, or both. | 1 |
| **ABSTRACT** | | |  |
| Structured summary | 2 | Provide a structured summary including, as applicable: background; objectives; data sources; study eligibility criteria, participants, and interventions; study appraisal and synthesis methods; results; limitations; conclusions and implications of key findings; systematic review registration number. | 2 |
| **INTRODUCTION** | | |  |
| Rationale | 3 | Describe the rationale for the review in the context of what is already known. | 3 |
| Objectives | 4 | Provide an explicit statement of questions being addressed with reference to participants, interventions, comparisons, outcomes, and study design (PICOS). | 3 |
| **METHODS** | | |  |
| Protocol and registration | 5 | Indicate if a review protocol exists, if and where it can be accessed (e.g., Web address), and, if available, provide registration information including registration number. | 4 |
| Eligibility criteria | 6 | Specify study characteristics (e.g., PICOS, length of follow-up) and report characteristics (e.g., years considered, language, publication status) used as criteria for eligibility, giving rationale. | 4 |
| Information sources | 7 | Describe all information sources (e.g., databases with dates of coverage, contact with study authors to identify additional studies) in the search and date last searched. | 4 |
| Search | 8 | Present full electronic search strategy for at least one database, including any limits used, such that it could be repeated. | Supplementary Table 2 |
| Study selection | 9 | State the process for selecting studies (i.e., screening, eligibility, included in systematic review, and, if applicable, included in the meta-analysis). | 4 |
| Data collection process | 10 | Describe method of data extraction from reports (e.g., piloted forms, independently, in duplicate) and any processes for obtaining and confirming data from investigators. | 5 |
| Data items | 11 | List and define all variables for which data were sought (e.g., PICOS, funding sources) and any assumptions and simplifications made. | 5 |
| Risk of bias in individual studies | 12 | Describe methods used for assessing risk of bias of individual studies (including specification of whether this was done at the study or outcome level), and how this information is to be used in any data synthesis. | 5 |
| Summary measures | 13 | State the principal summary measures (e.g., risk ratio, difference in means). | 5 |
| Synthesis of results | 14 | Describe the methods of handling data and combining results of studies, if done, including measures of consistency (e.g., I^2^) for each meta-analysis. | 5 |
| Section/topic | # | Checklist item |  |
| Risk of bias across studies | 15 | Specify any assessment of risk of bias that may affect the cumulative evidence (e.g., publication bias, selective reporting within studies). | 6 |
| Additional analyses | 16 | Describe methods of additional analyses (e.g., sensitivity or subgroup analyses, meta-regression), if done, indicating which were pre-specified. | N/A |
| **RESULTS** | | |  |
| Study selection | 17 | Give numbers of studies screened, assessed for eligibility, and included in the review, with reasons for exclusions at each stage, ideally with a flow diagram. | 7 |
| Study characteristics | 18 | For each study, present characteristics for which data were extracted (e.g., study size, PICOS, follow-up period) and provide the citations. | 7 |
| Risk of bias within studies | 19 | Present data on risk of bias of each study and, if available, any outcome level assessment (see item 12). | 7,8 |
| Results of individual studies | 20 | For all outcomes considered (benefits or harms), present, for each study: (a) simple summary data for each intervention group (b) effect estimates and confidence intervals, ideally with a forest plot. | 7,8 |
| Synthesis of results | 21 | Present results of each meta-analysis done, including confidence intervals and measures of consistency. | 7,8 |
| Risk of bias across studies | 22 | Present results of any assessment of risk of bias across studies (see Item 15). | 7,8 |
| Additional analysis | 23 | Give results of additional analyses, if done (e.g., sensitivity or subgroup analyses, meta-regression [see Item 16]). | N/A |
| **DISCUSSION** | | |  |
| Summary of evidence | 24 | Summarize the main findings including the strength of evidence for each main outcome; consider their relevance to key groups (e.g., healthcare providers, users, and policy makers). | 11 |
| Limitations | 25 | Discuss limitations at study and outcome level (e.g., risk of bias), and at review-level (e.g., incomplete retrieval of identified research, reporting bias). | 11 |
| Conclusions | 26 | Provide a general interpretation of the results in the context of other evidence, and implications for future research. | 12 |
| **FUNDING** | | |  |
| Funding | 27 | Describe sources of funding for the systematic review and other support (e.g., supply of data); role of funders for the systematic review. | 14 |

**Supplementary Table 3**: Summary of each anticholinergic burden measure. *Indicates country from which the list of medications included in the measure originates, e.g. the country in which patient drug charts or prescribing records were analysed. ‡Excluding medications determined to have no anticholinergic activity (score = 0), where applicable. ACB: Anticholinergic burden; ACBS = Anticholinergic Cognitive Burden Scale; ADS = Anticholinergic Drug Scale; ARS = Anticholinergic Risk Scale; CrAS = Clinician-rated Anticholinergic Scale; MARANTE = Muscarinic Acetylcholinergic Receptor Antagonist Exposure Scale; SAA = Serum Anticholinergic Activity

| **Anticholinergic medication measure** | **Country of origin** | **Number of anticholinergic medications listed** | **Scoring system** | **Basis of methodology** |
| --- | --- | --- | --- | --- |
| ACBS, Boustani 2008 | USA | 88 | Scale from 0 to 3 | Existing literature and expert opinion |
| ADS, Carnahan 2006 | USA | 117 | Scale from 0 to 3 | Existing literature |
| ARS, Rudolph 2008 | USA | 49 | Scale from 0 to 3 | Extensive literature review and expert opinion |
| Chew’s list, Chew 2008 | USA | 39 | Scale of 0, 0/+, +, ++ or +++ | SAA data |
| CrAS, Han 2008 | USA | 60 | Scale from 0 to 4 | Existing literature and expert opinion |
| Drug Burden Index (anticholinergic component), Hilmer 2007 | USA | Not specified | Dose-dependent | Study assessing ACB and physical/cognitive function in Medicare recipients |
| Duran’s list, Duran 2013 | Mixed | 100 | Scale from 0 to 2 | Systematic review of previously available ACB measures |
| MARANTE, Klamer 2017 | Belgium | 41 | Score of 1 or 2 | Existing literature, expert opinion and daily dosage calculations |
